# Improved orthologous databases to ease protozoan targets inference

**DOI:** 10.1186/s13071-015-1090-0

**Published:** 2015-09-29

**Authors:** Nelson Kotowski, Rodrigo Jardim, Alberto M. R. Dávila

**Affiliations:** Computational and Systems Biology Laboratory, Oswaldo Cruz Institute, FIOCRUZ, Avenida Brasil, 4365, 21040-360 Rio de Janeiro, RJ Brazil

**Keywords:** Comparative genomics, Homology inference, Target identification, Protozoa, Orthologous database, Distant homology, *Leishmania*, *Cryptosporidium*, *Entamoeba*

## Abstract

**Background:**

Homology inference helps on identifying similarities, as well as differences among organisms, which provides a better insight on how closely related one might be to another. In addition, comparative genomics pipelines are widely adopted tools designed using different bioinformatics applications and algorithms. In this article, we propose a methodology to build improved orthologous databases with the potential to aid on protozoan target identification, one of the many tasks which benefit from comparative genomics tools.

**Methods:**

Our analyses are based on OrthoSearch, a comparative genomics pipeline originally designed to infer orthologs through protein-profile comparison, supported by an HMM, reciprocal best hits based approach. Our methodology allows OrthoSearch to confront two orthologous databases and to generate an improved new one. Such can be later used to infer potential protozoan targets through a similarity analysis against the human genome.

**Results:**

The protein sequences of *Cryptosporidium hominis*, *Entamoeba histolytica* and *Leishmania infantum* genomes were comparatively analyzed against three orthologous databases: (i) EggNOG KOG, (ii) ProtozoaDB and (iii) Kegg Orthology (KO). That allowed us to create two new orthologous databases, “KO + EggNOG KOG” and “KO + EggNOG KOG + ProtozoaDB”, with 16,938 and 27,701 orthologous groups, respectively.

Such new orthologous databases were used for a regular OrthoSearch run. By confronting “KO + EggNOG KOG” and “KO + EggNOG KOG + ProtozoaDB” databases and protozoan species we were able to detect the following total of orthologous groups and coverage (relation between the inferred orthologous groups and the species total number of proteins): *Cryptosporidium hominis*: 1,821 (11 %) and 3,254 (12 %); *Entamoeba histolytica*: 2,245 (13 %) and 5,305 (19 %); *Leishmania infantum*: 2,702 (16 %) and 4,760 (17 %).

Using our HMM-based methodology and the largest created orthologous database, it was possible to infer 13 orthologous groups which represent potential protozoan targets; these were found because of our distant homology approach.

We also provide the number of species-specific, pair-to-pair and core groups from such analyses, depicted in Venn diagrams.

**Conclusions:**

The orthologous databases generated by our HMM-based methodology provide a broader dataset, with larger amounts of orthologous groups when compared to the original databases used as input. Those may be used for several homology inference analyses, annotation tasks and protozoan targets identification.

**Electronic supplementary material:**

The online version of this article (doi:10.1186/s13071-015-1090-0) contains supplementary material, which is available to authorized users.

## Background

Historically, the very definition of a Protozoa represents an open debate. Despite many classifications and changes provided over history [1–4], in this article we will refer to Protozoa as eukaryotic organisms, apart from those who do not have a primitive mitochondria, peroxisomes (*Archezoa*) and the shared characteristics which define the *Animalia*, *Fungi*, *Plantae* and *Chromista* kingdoms [4].

There are over 200,000 described protozoan organisms and among them over 10,000 parasites of invertebrate organisms and nearly all vertebrate ones [1]. There are several protozoan related diseases, which affect more than 25 % of the world population, such as Chagas’ disease, Human African Trypanosomosis, Leishmaniosis, Amoebiosis, Giardiosis, Toxoplasmosis, Cryptosporidiosis, Theileriosis, Babesiosis among many others [5–10].

Neglected Tropical Diseases (NTDs) are diseases caused by a variety of organisms and are usually associated to developing countries, which suffer from poor sanitation, hygiene, social and financial conditions. Over 1 billion people are affected by such diseases, in 149 countries worldwide [11]. Among the 17 NTDs listed by WHO, three are caused by protozoan organisms: Chagas’ disease (*Trypanosoma cruzi*), Human African Trypanosomosis (*Trypanosoma brucei*) and Leishmaniosis (*Leishmania* spp.) [11].

According to the 3^rd^ WHO report on NTDs, even though several advances have been achieved in the recent years, there is a permanent need for research and innovation in improved diagnosis, next-generation treatments and interventions for such NTDs [12, 13].

Leishmaniosis is a neglected disease caused by the *Leishmania* spp*.* and transmitted by phlebotomine sandflies [14]. More than 1.3 million people are infected worldwide, especially those who live in poor sanitation, hygiene and social conditions and WHO estimates about 20,000 to 30,000 deaths occur yearly [14]. Such disease has three distinct presentations: cutaneous, visceral and mucocutaneous, each of them related to different *Leishmania* spp. and world regions.

Leishmaniosis is hard to diagnose and treat. So far, the available drugs and vaccines are either toxic or present poor efficiency [15]. Also, its elevated treatment cost (up to US$252/patient, depending on the applied treatment and drugs) eventually becomes prohibitive to the most affected and poor countries [15].

Besides allowing for a better comprehension of such Protozoa organism, many molecular studies have been done over the last few years using DNA/RNA sequencing methodologies [16–18], which have been used in order to infer new drug targets. These data are available in several public databases and allow for comparative genomics studies among either closely or distant related organisms. Also, that might increase the odds of discovering relevant information applied to drug manufacturing or reuse, which could be later applied to disease treatments.

Comparative genomics mainly refers to homology and evolutionary dynamics between organisms, genes and proteins, which provides better understanding on how species evolved through comparing either their complete genomes or specific genes [19]. Homologous genes share a common ancestry, either intra- or inter-species. Several scenarios relate to homology, such as orthology, paralogy, horizontal gene transfer, gene loss, orphan genes and others; for this study, we will focus on orthology aspects only [20].

Orthology might be inferred when the same genes or proteins are present in distinct species, and this was due to a speciation event [20].

Homology inference has become an important issue when inferring function to recently sequenced genes because orthologs tend to preserve their ancestor function. Besides that, such studies provide a better insight on genes evolutionary history and consequently, to the species evolution [20, 21].

Inferring putative function is one of the particular benefits in orthologous group (OG) assignment, especially when dealing with recently sequenced genome data [22]. In addition, OGs may provide us a better comprehension on species evolutionary relationships [23], since it is through such data that one might provide information that could help on both evolutionary and functional analysis [24].

Moreover, several tasks could benefit from OGs, such as genome annotation, gene conservation, protein family identification, phylogenetic tree reconstruction, pharmacology and many others [22, 24–27]. Topics as positional orthology and synteny conservation among orthologs are also appealing to those who aggregate genomic context in their homology inference methods [28].

There are several available methodologies to aid on homology detection. Besides a simple categorization effort [26], we will follow Dalquen’s proposition [29]. Briefly, three distinct approaches are available: (i) the one which use multiple sequence alignment (MSA) scores along with reciprocal best hits, such as OrthoSearch [30], OrthoMCL [24] and InParanoid [31]; (ii) that which rely on evolutionary distance calculus, as RSD [32, 33]; (iii) and that based on phylogenetic trees reconstruction, as SPIMAP [34].

Many orthologous databases (OD) are created by homology inference methods. This is the case for OrthoMCLDB [35]; InParanoid [36]; Roundup [32]; COG/KOG [37] and EggNOG [38, 39].

OrthoSearch [40] is a scientific workflow [41] for homology inference among species. Initially conceived as a Perl-based routine, it uses a reciprocal best hits, HMM-based approach. OrthoSearch has already proven to be effective inferring orthology among five protozoan genomes, using COG and KOG ODs [27].

In this work, we propose an update and a new functionality for OrthoSearch, showing it as an effective tool in providing means to create new ODs (n-ODs). So far, we tested our methodology in a controlled, three steps scenario: (i) Protozoa orthology inference and (ii) n-ODs creation, both supported by publicly available ODs used as input; and (iii) improved Protozoa orthology inference, supported by such recently created n-ODs.

With our methodology and generated n-ODs, we expect to be able to provide ODs with broader data sets, which in turn can be applied in target identification for protozoan organisms, such as stated by Timmers *et al.* [42] review on research efforts related to genomic database development for protozoan parasites.

Moreover, previous initiatives, such as the study performed by Tschoeke *et al.* [18] regarding the *Leishmania amazonensis* parasite, as well as the *Leishmania donovani* comparative genomics analysis performed by Satheesh *et al.* [43] corroborate the benefits provided by the use of broader orthologous data sets.

## Methods

### OrthoSearch improvements and analyses scenarios

In order to reach our main methodological goal, which is to provide OrthoSearch with means to create n-ODs, we revisited its original pipeline. Notably (i) we adopted HMMER version 3 and (ii) changed from a Perl-based routine to C++ 4.43 and Ruby 1.8.7 modules. A dedicated Ubuntu 12.04 single-server machine with 64 cores and 32GB RAM was used for all assembled scenarios.

### OrthoSearch for protozoa orthology inference

OrthoSearch needs as input data an (i) OD and (ii) an organism multifasta protein data. We used Kegg Orthology (KO) [44, 45], EggNOG KOG and ProtozoaDB as input ODs. KO, downloaded via FTP, contains data from all life domains – Archaea, Bacteria and Eukarya. EggNOG KOG is a eukaryotic-only groups Eggnog subset [39], downloaded directly from its website. ProtozoaDB OGs [46] (which contain only protozoan species) were also used for our analyses. Details about each OD are available in Additional file 1.

We randomly selected three protozoan species as OrthoSearch organisms input data: *Cryptosporidium hominis*, *Entamoeba histolytica* and *Leishmania infantum,* each with 3,885, 7,973 and 7,872 proteins and downloaded such data from ProtozoaDB [46].

### OrthoSearch for orthologous database building

**Fig. 1:**
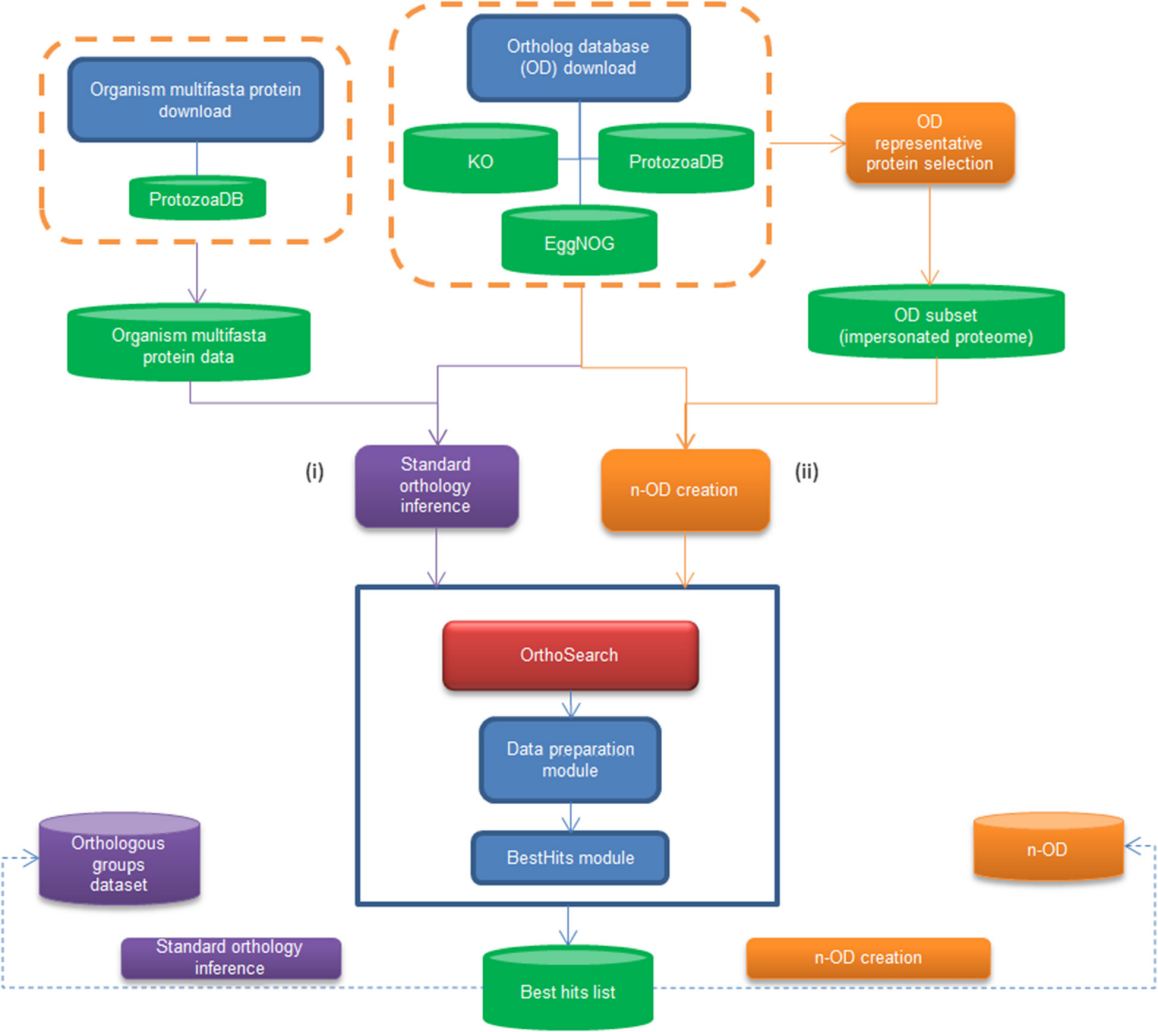
OrthoSearch pipeline and the two possible strategies; This figure describes how OrthoSearch pipeline works in a (i) standard orthology inference; and how it is reused to (ii) create a n-OD

Figure 1 depicts OrthoSearch pipeline with its two possibilities, which could be (i) a standard orthology inference or (ii) a n-OD creation. In order to build the n-ODs, we used as input data an OD in its original composition; and another OD data subset, which enacts as an organism multifasta protein data.

That was called an impersonated proteome and was generated by choosing a representative protein for each OG at the confronted OD. The selection and extraction of such proteins was performed with the support of a Python script kindly developed by Salvador Capella (personal communication, URL: https://github.com/scapella/trimal/blob/dev/scripts/get_sequence_representative_from_alignment.py) and an internally developed Ruby script. Each representative protein identifier and its amino acid sequence were stored in a single multifasta file (see Additional file 2).

We started with KO as our fixed, complete OD, against an impersonated EggNOG KOG multifasta protein data. Reciprocal best hits between both of them were processed using internal scripts developed in both Ruby and Unix/POSIX shell script languages, so that, new OGs were created and arranged, generating the n-OD conveniently named “KO + EggNOG KOG”.

During such n-OD creation, there were three possible scenarios related to the fixed database OGs – KO – and the impersonated proteome. Briefly, (i) those OGs from KO database which did not have any reciprocal best hit with the impersonated OGs from EggNOG KOG; and (ii) those OGs from EggNOG KOG that did not have any reciprocal best hit against KO database were identified, selected and incorporated to our n-OD (“KO + EggNOG KOG”), without any changes; (iii) those OGs from KO database which presented a reciprocal best hit with the impersonated OGs from EggNOG KOG were expanded, by adding up every respective EggNOG KOG OG proteins into such KO group.

Therefore, at the end of this run, we had a n-OD called “KO + EggNOG KOG” which comprised original, unaltered KO and EggNOG KOG OGs, as well as expanded KO OGs, which now also contain EggNOG KOG protein data. Once such n-OD was created, we performed the same steps described above, confronting “KO + EggNOG KOG” against ProtozoaDB impersonated OGs, which generated our second n-OD called “KO + EggNOG KOG + ProtozoaDB”.

### Inferring protozoan orthologs with OrthoSearch and the n-ODs

Having built the two n-ODs: “KO + EggNOG KOG” and “KO + EggNOG KOG + ProtozoaDB”, we analyzed OrthoSearch in a standard orthology inference against the three above mentioned protozoan species.

### Comparison between the n-ODs and OrthoMCLDB

The three protozoan species were confronted against OrthoMCLDB through online phyletic pattern search queries, in order to infer its orthologous proteins, in the same way as we did with the n-ODs created by our proposed methodology.

Quantitative results obtained while executing both OrthoSearch (with the two n-ODs) and OrthoMCLDB against the three protozoan species were compared in order to offer a better understanding of the proposed methodology behavior and to analyze if we were able to provide better results or not.

### Potential *Leishmania* spp. targets against the human proteome

In order to identify potential protozoan targets that are not available at the human genome, a BlastP [47] was performed between the largest n-OD generated by our methodology - “KO + Eggnog KOG + ProtozoaDB” (details on the orthologous groups proteins are available at Additional file 3) and the human proteome, downloaded via RefSeq [48]. We used BlastP 2.2.28+ with 0.1 as e-value, extracted and analyzed the orthologous groups which did not perform any hit against the human proteome but provided results against *Leishmania* spp. and therefore could represent potential targets. A BlastP was also performed against KO, Eggnog KOG and ProtozoaDB orthologous databases separately.

## Results

### OrthoSearch for protozoa orthology inference

**Fig. 2:**
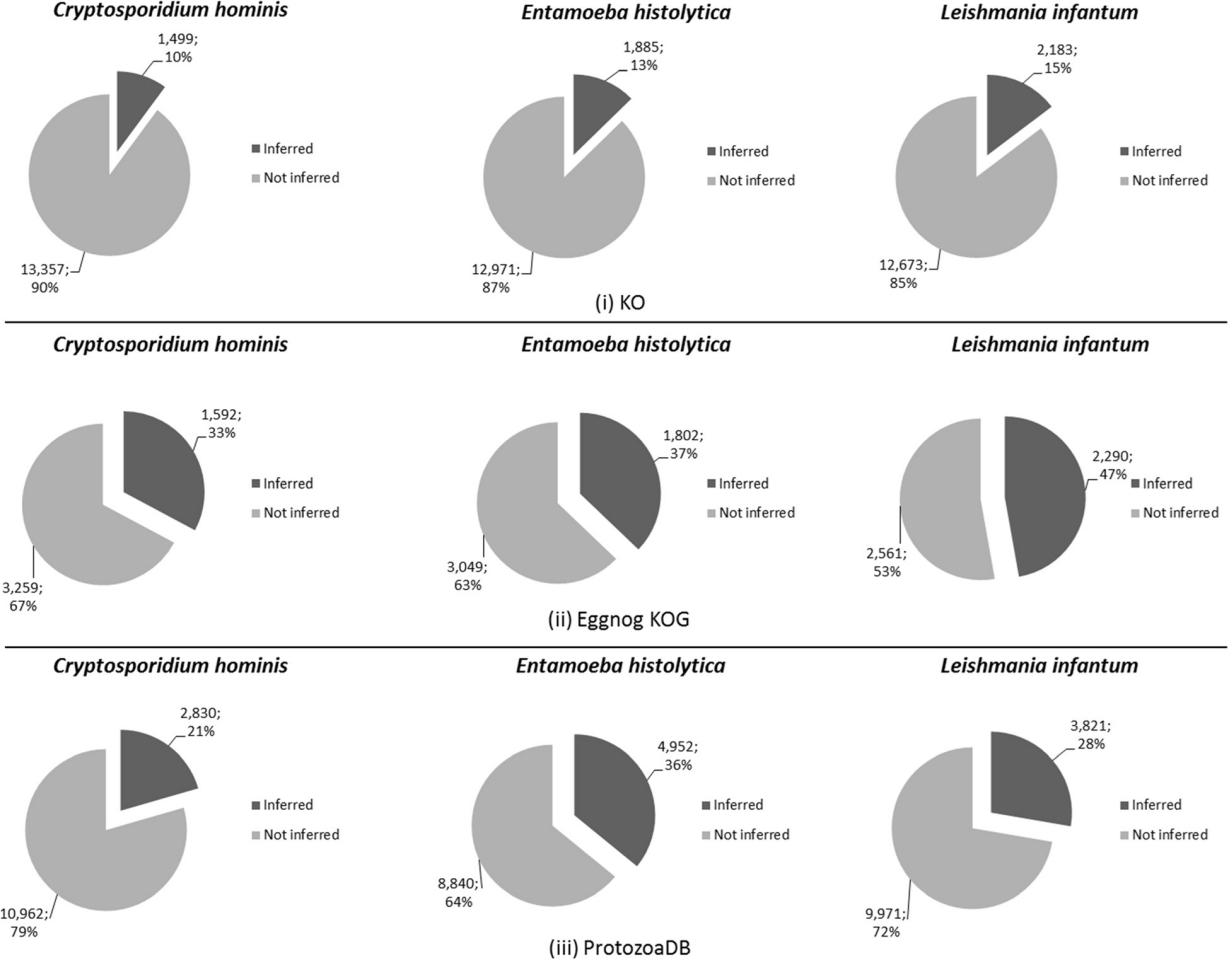
OrthoSearch inferred orthologous groups and coverage per organism; A detailed view on how many orthologous groups were inferred with (i) KO, (ii) EggNOG KOG and (iii) ProtozoaDB databases and what do such numbers represent against the organisms total protein numbers

The protein data of the three protozoan species were confronted against (i) KO, (ii) EggNOG KOG and (iii) ProtozoaDB ODs (Fig. 2). ProtozoaDB performed best, with: 2,830 OGs against *Cryptosporidium hominis*, 4,952 for *Entamoeba histolytica* and 3,821 for *Leishmania infantum*.

With such data, we extracted coverage percentage information, which shows the total number of OGs inferred by OrthoSearch versus how many OGs are contained within each OD. For *Cryptosporidium hominis*, which has the smallest number of proteins of the three protozoan species studied, EggNOG KOG performed best, with 33 % coverage. *Entamoeba histolytica* also performed well with EggNOG KOG (37 %), but showed very similar results with ProtozoaDB (36 %), while showing a poor coverage with KO (13 %). Finally, *Leishmania infantum* had the best coverage (47 %), with EggNOG KOG.

**Fig. 3:**
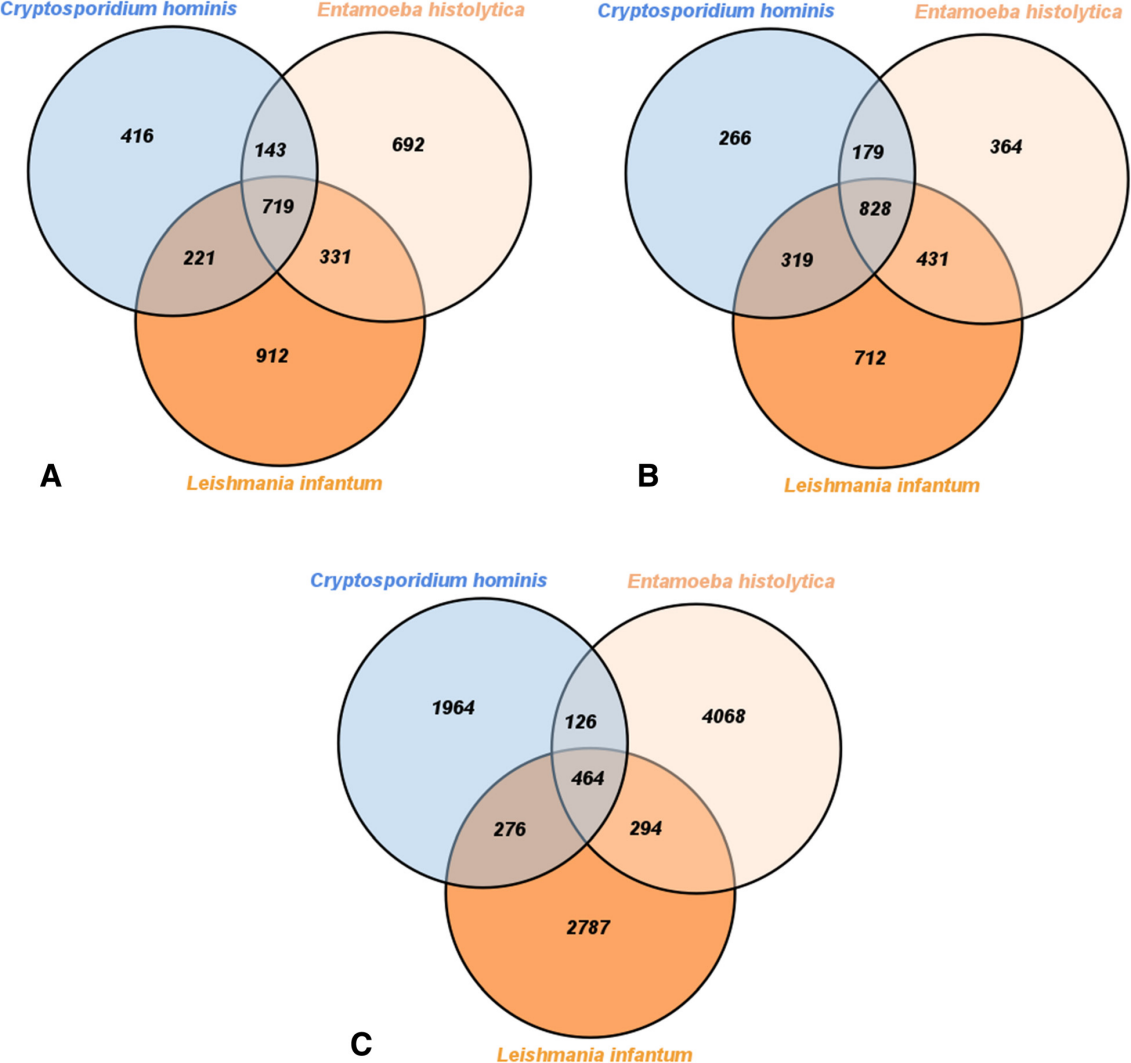
Orthologous groups’ superposition; A Venn Diagram depicting how many species-specific, pairwise and core orthologous groups were inferred for the protozoan organisms against (**a**) KO, (**b**) EggNOG KOG and (**c**) ProtozoaDB databases

Internal scripts, developed with the R language and its Venn Diagram library, processed reciprocal best hits for such protozoan species. We identified species-specific, pair-to-pair and core OGs, depicted at Fig. 3.

EggNOG KOG presented the best core (ratio between OGs shared by the three protozoan species and all the inferred OGs), corresponding to 26.72 % of the total inferred best hits (828/3,099 OGs), followed by KO - 20.94 % (719/3,434 OGs) and ProtozoaDB - 4.65 % (464/9,979 OGs).

In addition, ProtozoaDB presented the best species-specific results, with *Entamoeba histolytica* performing 40.77 % of the total OGs (4,086/9,979); *Leishmania infantum* with 27.93 % (2,787/9,979); and *Cryptosporidium hominis* with 19.68 % (1,964/9,979).

### OrthoSearch for Orthologous Database building

Table 1 shows details on how many OGs remained intact and directly migrated to the n-ODs created by our methodology as well as those that were expanded.

**Table 1:** n-OD creation details

n-OD creation step		OGs in the OD (i)	OGs in the impersonated proteome (ii)	Intact OGs on the OD(iii)	Intact OGs in the impersonated proteome (iv)	OGs to be expanded(v)	Total OGs in the n-OD (vii)
OD name	Impersonated proteome						
KO	EggNOG KOG	14856	4851	12087	2082	2769	16938
KO + EggNOG KOG	ProtozoaDB	16938	13792	13909	10763	3029	27701

After “KO + EggNOG KOG” building, we had a 14.02 % increase in the total number of OGs when compared to KO (16,938/14,856 OGs). In addition, 18.63 % KO OGs were expanded (2,769/14,856 OGs). When this n-OD was confronted against the impersonated ProtozoaDB OGs, we had a 63.54 % increase in the total number of OGs (27,701/16,938) and 17.88 % “KO + Eggnog KOG” OGs were expanded. Table 2 summarizes how many OGs, proteins, the average proteins per group and each n-OD size. “KO + EggNOG KOG + ProtozoaDB”, when compared to KO, provides 86.46 % more OGs (27,701/14,856 OGs) and 22.45 % more proteins (2,582,631/2,109,027 proteins). At last, Table 3 shows protozoan species representation at each created n-OD.

**Table 2:** n-ODs main characteristics

OD/feature	Total OGs	Total proteins	Average proteins per OG	OD size
KO + EggNOG KOG	16938	2518449	149	1.4 GBytes
KO + EggNOG KOG + ProtozoaDB	27701	2582631	93	1.5 GBytes

**Table 3:** Protozoan species contribution for each n-OD

OD	Total OGs	OGs with at least one protozoan species	Ratio	Total OD proteins	Protozoan proteins	Ratio
KO	14856	3612	24.31 %	2108653	46027	2.18 %
KO + EggNOG KOG	16938	5851	34.54 %	2518449	74630	2.96 %
KO + EggNOG KOG + ProtozoaDB	27701	17305	62.47 %	2582631	138814	5.37 %

### Inferring protozoan orthologs with OrthoSearch and the n-ODs

**Fig. 4:**
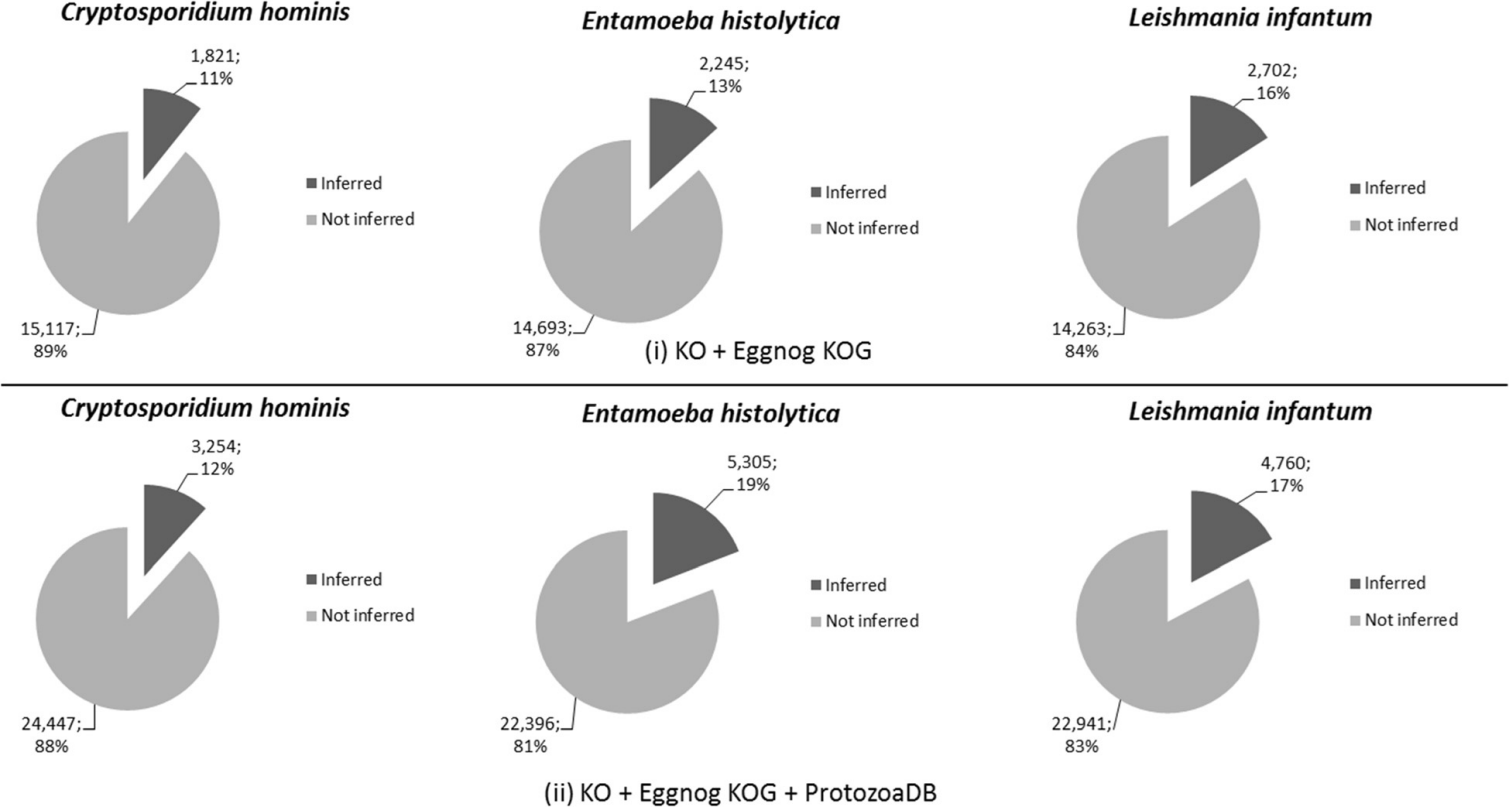
OrthoSearch inferred orthologous groups and coverage, per organism, with the databases created by the methodology itself; A detailed view on how many orthologous groups were inferred with (i) “KO + EggNOG KOG” and (ii) “KO + Eggnog KOG + ProtozoaDB” databases and what do such numbers represent against the organisms total protein numbers

With these recently created n-ODs, on our second scenario we executed OrthoSearch using as input such n-ODs and the same three protozoan species then compared the obtained results against previous KO analysis. Figure 4 depicts coverage percentage data for each of the OG databases created by the methodology itself, for each organism adopted.

Our methodology provided an 86.47 % increase on the total number of OGs and a 22.45 % on the total amount of proteins when comparing “KO + EggNOG KOG + ProtozoaDB” against KO. Although there was a relevant increase in the number of inferred OGs for *Cryptosporidium hominis* (from 1,499 up to 3,254 groups), coverage increase was very subtle (10 %-12 %). *Entamoeba histolytica*, on the other hand, shows a relevant increase in coverage (19 %), especially when confronted against “KO + EggNOG KOG + ProtozoaDB” n-OD. *Leishmania infantum* had a very similar behavior to *Cryptosporidium hominis*, with a total of up to 4,760 OGs and from 15 % up to 17 % coverage.

**Fig. 5:**
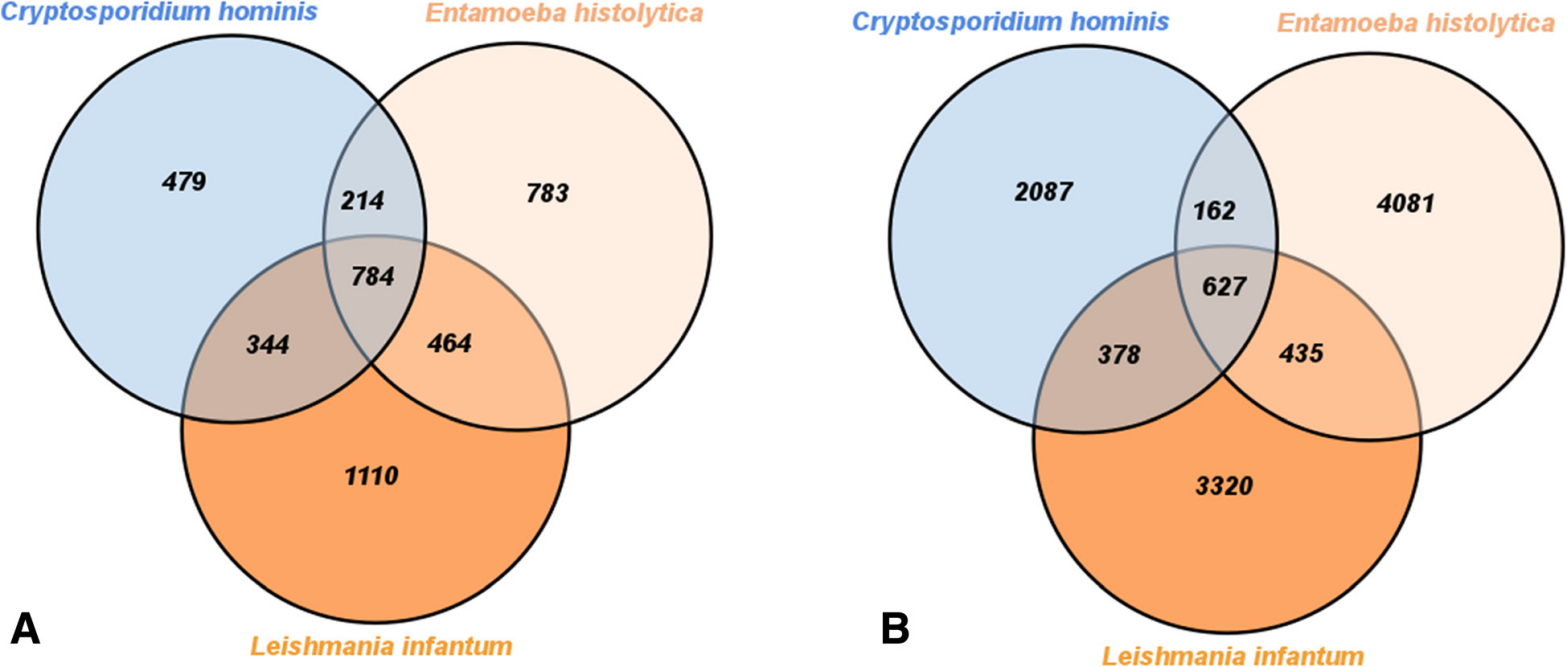
Orthologous groups’ superposition; A Venn Diagram depicting how many species-specific, pairwise and core orthologous groups were inferred for the protozoan organisms against (**a**) “KO + EggNOG KOG” and (**b**) “KO + EggNOG KOG + ProtozoaDB” n-ODs

We also consolidated the reciprocal best hits obtained in Venn diagrams, so that we might have a glimpse on species-specific, pair-to-pair and core OGs, as shown in Fig. 5.

Additional file 4 shows how many OGs were inferred as best hits with OrthoSearch when protozoan species were confronted against each of the generated n-ODs. “KO + EggNOG KOG” provided the best OGs core coverage ratio – 18.76 % (784/4,178) and “KO + EggNOG KOG + ProtozoaDB”, 5.65 % (627/11,089). “KO + EggNOG KOG + ProtozoaDB” had the best results in species-specific OGs, with *Entamoeba histolytica* at a 36.80 % ratio (4,081/11,089); *Leishmania infantum* with 29.94 % (3,320/11,089); and *Cryptosporidium hominis* at 18.81 % (2,086/11,089).

### Comparison between the n-ODs and OrthoMCLDB

After submitting the same protozoan species to OrthoMCLDB online phyletic pattern search, 3,516 (*Cryptosporidium hominis*), 6,107 (*Entamoeba histolytica*) and 7,538 (*Leishmania infantum*) OGs were inferred. OrthoMCLDB inferred a 760 OGs core, which represents 5.12 % of the total best hits (760/14,846).

**Fig. 6:**
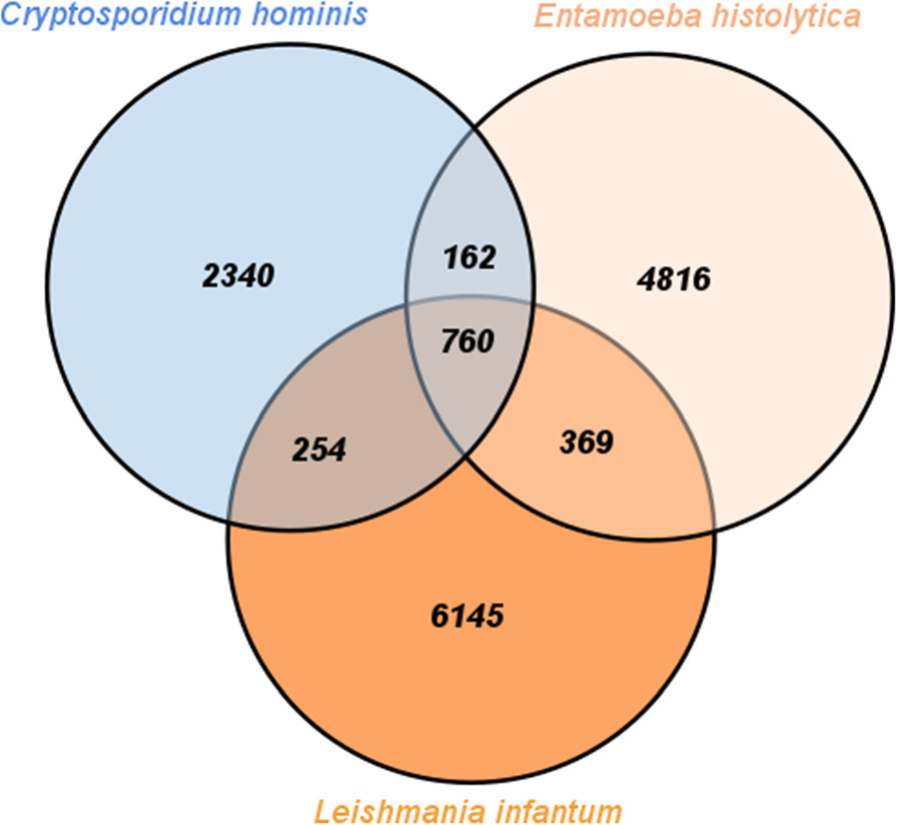
Orthologous groups’ superposition; A Venn Diagram depicting how many species-specific, pairwise and core orthologous groups were inferred for the protozoan organisms against OrthoMCLDB

Concerning species-specific OGs, OrthoMCLDB detected 2,340 – 15.76 % (2,340/14,846) OGs for *Cryptosporidium hominis;* 4,816 – 32.44 % (4,816/14,846) for *Entamoeba histolytica*; and 6,145 – 41.39 % for *Leishmania infantum*; at last, pairwise shared OGs corresponded to 162 (*Cryptosporidium hominis* and *Entamoeba histolytica*), 254 (*Cryptosporidium hominis* and *Leishmania infantum*) and 369 (*Entamoeba histolytica* and *Leishmania infantum*) OGs respectively. Figure 6 shows a Venn diagram with obtained results.

### Potential *Leishmania* spp. targets against the human genome

A BlastP against our largest created n-OD, “KO + Eggnog KOG + ProtozoaDB” (27,701 orthologous groups) allowed us to infer 7,622 (27.5 %) orthologous groups which did not perform any hit against the human proteome. Among such, 6.5 % (1,805/27,701) groups belong to KO or Eggnog KOG, but are not available in ProtozoaDB, which contains only protozoan organisms (*Leishmania* spp. included). Furthermore, 13 orthologous groups (0.05 %) contain at least one *Leishmania* spp*.* (Table 4), that should be considered as potential targets for further analysis.

**Table 4:** Potential *Leishmania* spp. targets

Orthologous group	Annotation
K14118.cdhit	Energy-converting hydrogenase B subunit I
K13666.cdhit	UDP-GlcNAc:polypeptide alpha-N-acetylglucosaminyltransferase
K03668.cdhit	Heat shock protein HslJ
K08907.cdhit	Light-harvesting complex I chlorophyll a/b binding protein 1
K13690.cdhit	Alpha-1,3-mannosyltransferase
K13674.cdhit	Phosphoglycan alpha-1,2-arabinopyranosyltransferase
K08276.cdhit	Ecotin
K02817.cdhit	PTS system, trehalose-specific IIB component
K01833.cdhit	Trypanothione synthetase/amidase
K03329.cdhit	Hypothetical protein
K01973.cdhit	Mitochondrial RNA editing ligase 1
K03356.cdhit	Anaphase-promoting complex subunit 9
K13672.cdhit	Galactofuranosyltransferase

The same BlastP query against each of the original ODs provided us the results listed in Table 5. These groups have no similarity with the human proteome and have at least one *Leishmania* spp*.* sequence.

**Table 5:** *Leishmania* spp. orthologous groups with no hit against the human proteome (Original orthologous databases)

Orthologous database	Orthologous groups
KO	5
Eggnog KOG	1
ProtozoaDB	1524

## Discussion

In this analysis, we adopted new programming languages and updated the OrthoSearch pipeline with several bioinformatics tools, rewriting it to be later used in homology inference analyses and n-ODs creation.

OrthoSearch uses an algorithm based on reciprocal best hits calculation via HMM profiles, with Mafft being responsible for providing MSA’s and HMMER3 tools for generating HMM models, profiles and statistics. It benefits from multithreading provided by both Mafft and HMMER3 and accepts any of both tools available parameters. In a regular run, OrthoSearch uses an e-value cut-off flexible enough in order to aid on later profiles calculation.

Each of the studied ODs has its particular characteristics (e.g., while KO contains OGs from all life domains, EggNOG KOG contains only eukaryotes OGs and ProtozoaDB only protozoan OGs). That might influence the obtained results when running OrthoSearch with such ODs and organisms.

OrthoSearch execution with KO OD provides a significantly small core compared to KO size and the total number of best hits. That could be explained as KO contains proteins from many evolutionarily distant organisms, what could pose a challenge in the identification of closely related OGs.

Later, EggNOG KOG OD provided a discrete increase in the obtained protein core, most likely due to EggNOG KOG having only eukaryotic organisms’ data. While ProtozoaDB OD provided the smallest core among the three ODs, the total number of species-specific protein is extremely higher. This could be due to the reduced number of species in ProtozoaDB OD, along with the fact that all of those are protozoan organisms. Basically, the odds of obtaining a hit with a protein belonging to the own species being analyzed within OrthoSearch could increase.

We opted to choose a representative protein for each OG at the confronted OD and impersonate an organism multifasta protein data because that could minimize the required computational power and time needed to run OrthoSearch analyses.

Since an OD contains several OGs, which also contains several proteins, that would easily escalate the required time to confront such ODs. In addition, as each OG contains two or more proteins usually from closely related organisms, that could imply the possibility of two (or more) distinct proteins from the same OG obtaining a hit with distinct OGs at the confronted OD.

Our scenarios for n-OD creation were based on KO, EggNOG KOG and ProtozoaDB ODs. According to the literature, each of these ODs were created through particular methodologies: the use of metabolic pathways (KO), heuristic approaches and Gene Ontology [49] support (EggNOG KOG) and OrthoMCL algorithm (ProtozoaDB).

Our methodology allowed us to create n-ODs that either contain intact OGs which originated from the source or the confronted ODs or expanded OGs from the obtained reciprocal best hits inferred by OrthoSearch. The intact OGs contribution relates to offering more OGs for further analyses, while expanded ones provide more variability than those OGs from the original databases.

Besides providing a means to improve ProtozoaDB orthology inference, we opted to begin our n-OD creation tasks with KO and Eggnog due to both database variability – proteins from organisms from all life domains – and size. We also decided to maintain the original orthologous groups database identifiers, as well as their functional annotation. That might ease further steps related to information provenance.

Our proposed methodology works as a non-intrusive approach to a HMM-based pipeline – OrthoSearch – without changing its core functions. It uses ODs as input data and is capable to create n-ODs without requiring extensive computational power.

When looking at how protozoan species data fit to our proposed n-OD creation methodology, we observe a 61.98 % increase from KO to “KO + EggNOG KOG” OD, scaling from 3,612 up to 5,851 OGs with at least one Protozoa protein. In addition, the total number of protozoan proteins had a 162 % increase (from 46,027 up to 74,630) in such n-OD.

Furthermore, when KO and “KO + EggNOG KOG + ProtozoaDB” ODs were compared, we identified a 379 % increase in the number of OGs that contain at least one Protozoa protein (from 3,612 up to 17,305) and a 300 % increase in the total number of protozoan proteins (from 46,027 up to 138,814).

A broader dataset is usually desirable, as it may increase the odds of obtaining hits while inferring homology. As more organisms contribute to a n-OD, one might be able to obtain more hits with regular OrthoSearch runs confronting n-ODs and organisms multifasta protein data.

OrthoSearch uses a Markov chain based approach in order to create the n-OD OGs, which tend to comprise more evolutionary distant orthologous proteins than BLAST-based methodologies, such as OrthoMCLDB.

With the n-ODs created within our methodology, our initiative is another step to reinforce possibilities to build a gold, reference dataset [50] for orthology inference.

As more OGs (and respective proteins) from distinct species are added up to the n-ODs created, the methodology offers a broader dataset, with more data variability. Such data may be used for further homology inference analyses, which is a very desirable aspect in several comparative genomics applications. For example, phylogenomic studies which try to address gene conflicts and allow for optimal tree construction [51] or even review species’ definition [52].

The obtained results point towards the success of our proposed methodology, which encourage us in refining and creating more n-ODs. Such n-ODs might also be used in order to improve future functional annotation, re-annotation and also potential targets identification.

OrthoMCLDB was able to provide a broader species-specific OGs dataset, varying from 12 % *(Cryptosporidium hominis* - 2,340/2,086) up to 85 % *(Leishmania infantum* – 6,145/3,320) more OGs than our methodology. That may be due to several OrthoMCLDB groups containing only in-paralogs (20,853/124,740) from a single species lineage [35].

When looking at pairwise groups, there is a change in this scenario. Our methodology either provides the same quantitative results as OrthoMCLDB (*Cryptosporidium hominis* and *Entamoeba histolytica* – 162 OGs) or better, with 17.88 % more OGs (*Entamoeba histolytica* and *Leishmania infantum* – 435/369) and up to 48.81 % more OGs (*Cryptosporidium hominis* and *Leishmania infantum* – 378/254).

OrthoMCLDB inferred a larger absolute number of OGs in the three protozoan species core (760) than our methodology (627), which may be related to a broader seed of OGs (124,740/27,701). On the other hand, OrthoMCLDB performed poorly in coverage aspect, with only 0.06 % OGs in its core (760/124,740), while our methodology covered 2.26 % OGs (627/27,701), with our largest n-OD (“KO + EggNOG KOG + ProtozoaDB”). This may be due to the fact that the studied species are not so closely related. In addition, OrthoMCLDB uses a Blast-based algorithm, in a less sensitive approach than our methodology (OrthoSearch uses a protein-profile comparison) [9, 32].

Our methodology provides means for improved orthologous database creation using a HMM-based approach. Those new databases may contain a greater set of evolutionary distant homologous proteins, which could further extend the odds of inferring knowledge regarding the target organisms.

Specifically, our analyses allowed for a better comprehension on three protozoan species, as well as a deeper analysis on potential targets. For example, the obtained protozoan core orthologous proteins may allow us to evaluate which of these are housekeeping proteins and how they relate to the organism fitness.

Also, the species specific proteins – those which do not belong to the core, or those shared between two of the three studied protozoan organisms might be explored either as species-specific or group-specific targets, respectively.

The obtained BlastP results allowed us to infer orthologous groups which contain protozoan proteins – specifically *Leishmania* spp. - that could be used as potential targets for further analysis, as they posed no hit against the human proteome.

Among the *Leishmania* spp. inferred orthologous groups without hits against the human proteome (Table 4) are proteins already described in the literature as possible drug targets, briefly: trypanothione [53] (K01833.cdhit) – which relates to defense against oxidative stress [54]; and alpha-1,3-mannosyltransferase [55, 56] (K13690.cdhit) – enzyme essential to add mannose on the glycosylphosphatidyl, relates to the growing resistance to miltefosine. However, there are also other proteins, not yet described as drug targets, which should be further studied, briefly: the energy-converting hydrogenase B subunit I [55] (K14118.cdhit), found in the Archaea organism *Methanothermobacter thermautotrophicus*, which belongs to a domain related to MnhB subunit of Na+/H+ antiporter and is predicted as an integral membrane protein [57, 58]; and galactofuranosyltransferase [59] (K13672.cdhit), related to the LPG1 gene, which acts as a major ligand for macrophage adhesion [60, 61].

Our methodology also provided means to allocate new and evolutionary distant proteins to the original orthologous groups’ databases, identifying orthology relationships which have not been previously described.

Even though this is a preliminary analysis, it allowed us to evaluate the applied methodology and to forecast how its results may be used for protozoan target identification, either in a species-specific or shared point-of-view. This methodology will be later applied to all of 22 ProtozoaDB [46] protozoan organisms.

## Conclusions

Our analyses used initially KO and EggNOG KOG databases as a starting point, adding later ProtozoaDB. We were able to create two n-ODS, “KO + EggNOG KOG” and “KO + EggNOG KOG + ProtozoaDB”, with each providing a larger amount of OGs when compared to the original. Those represent a broader dataset that can be used in future homology inference, annotation transfer and protozoan targets identification.

So far, our methodology allowed the identification of 13 potential targets in protozoan that would not have been identified without the distant homology approach provided by OrthoSearch and the n-ODs created by our methodology.

## Additional files

Additional file 1:
**Orthologous databases main characteristics.** This file provides a detailed view on the orthologous databases characteristics, regarding: how many organisms (total and protozoan only), orthologous groups, total proteins and the database total size. (XLS 6 kb) Additional file 2:
**Sample impersonated multifasta protein sample; This file provides a quick view on how the organisms’ representative proteins were stored in a single multifasta file, which impersonates an organism proteome and actually contains proteins from several orthologous groups from a single orthologous database.** (FASTA 1 kb) Additional file 3:
**“KO + Eggnog KOG + ProtozoaDB” orthologous groups (protein identifiers only).** For each ortholog group, we list all protein identifiers (corresponding organism and accession number). (BZ2 9971 kb) Additional file 4:
**Inferred RBH between protozoan organisms and orthologous databases.** This file provides information on inferred RBH between protozoan species against KO database and the two n-ODs created by the methodology as well. It is detailed on species-specific, pairwise and core RBH (shared between all organisms) absolute RBH and their percentage when compared to the total amount of obtained RBH. (XLS 27 kb)

## Abbreviations

KO: Kegg Orthology
OG: orthologous group
OD: orthologous database
n-OD: new orthologous database

## References

[1] Imam T (2009). The complexities in the classification of protozoa: a challenge to parasitologists. Bayero J Pure Appl Sci.

[2] Cavalier-Smith T (2009). Predation and eukaryote cell origins: A coevolutionary perspective. Int J Biochem Cell Biol.

[3] Cavalier-Smith T. Kingdoms Protozoa and Chromista and the eozoan root of the eukaryotic tree. Biol Lett. 2009;6(3):rsbl20090948. 10.1098/rsbl.2009.0948 PMC2880060 20031978

[4] Cavalier-Smith T (1993). Kingdom Protozoa and Its 18 Phyla. Microbiol Rev.

[5] Widmer G, London E, Zhang L, Ge G, Tzipori S, Carlton J, et al. Preliminary Analysis of the Cryptosporidium muris Genome. In: Ortega-Pierres G, Cacciò Simone M, Fayer R, Mank TG, Smith HV, Thompson RCA, editors. GIARDIA AND CRYPTOSPORIDIUM From Molecules to Disease. Wallingford, UK: CAB International; 2009. p. 320–7.

[6] Thompson RCA. The Impact of Giardia on Science and Society. In: Ortega-Pierres, Guadalupe; Cacciò, Simone M; Fayer, Ronald; Mank, Theo G; Smith, Huw V; Thompson R, editors. GIARDIA AND CRYPTOSPORIDIUM From Molecules to Disease. Wallingford, UK: CAB International; 2009. p. 1–11.

[7] Pain A, Renauld H, Berriman M, Murphy L, Yeats C, Weir W, Kerhornou A, Aslett M, Bishop R, Bouchier C, Cochet M, Coulson RMR, Cronin A, de Villiers EP, Fraser A, Fosker N, Gardner M, Goble A, Griffiths-Jones S, Harris DE, Katzer F, Larke N, Lord A, Maser P, McKellar S, Mooney P, Morton F, Nene V, O’Neil S, Price C (2005). Genome of the host-cell transforming parasite Theileria annulata compared with T. parva. Science.

[8] Elmore S, Jones JL, Conrad P, Patton S, Lindsay DS, Dubey JP (2010). Toxoplasma gondii: epidemiology, feline clinical aspects, and prevention. Trends Parasitol.

[9] Carlton JM, Adams JH, Silva JC, Bidwell SL, Lorenzi H, Caler E, Crabtree J, Angiuoli SV, Merino EF, Amedeo P, Cheng Q, Coulson RMR, Crabb BS, Del Portillo HA, Essien K, Feldblyum TV, Fernandez-Becerra C, Gilson PR, Gueye AH, Guo X, Kang’a S, Kooij TWA, Korsinczky M, Meyer EV-S, Nene V, Paulsen I, White O, Ralph SA, Ren Q, Sargeant TJ (2008). Comparative genomics of the neglected human malaria parasite Plasmodium vivax. Nature.

[10] Brayton K, Lau AOT, Herndon DR, Hannick L, Kappmeyer LS, Berens SJ, Bidwell SL, Brown WC, Crabtree J, Fadrosh D, Feldblum T, Forberger H, Haas BJ, Howell JM, Khouri H, Koo H, Mann DJ, Norimine J, Paulsen IT, Radune D, Ren Q, Smith RK, Suarez CE, White O, Wortman JR, Knowles DP, McElwain TF, Nene VM (2007). Genome sequence of Babesia bovis and comparative analysis of apicomplexan hemoprotozoa. PLoS Pathog.

[11] WHO | Neglected Diseases [http://www.who.int/neglected_diseases/diseases/en/].

[12] WHO/Department of control of neglected tropical diseases. Investing to Overcome the Global Impact of Neglected Tropical Diseases. Geneva, Switzerland: World Health Organization; 2015.

[13] The London declaration on Neglected Tropical Diseases [http://unitingtocombatntds.org/sites/default/files/resource_file/london_declaration_on_ntds.pdf].

[14] WHO | Leishmaniasis [http://www.who.int/mediacentre/factsheets/fs375/en/].

[15] WHO Technical Report Series [http://whqlibdoc.who.int/trs/WHO_TRS_949_eng.pdf].

[16] Singh N, Chikara S, Sundar S (2013). SOLiD^TM^ sequencing of genomes of clinical isolates of leishmania donovani from india confirm leptomonas co-infection and raise some key questions. PLoS One.

[17] Brotherton M-C, Bourassa S, Leprohon P, Légaré D, Poirier GG, Droit A, Ouellette M (2013). Proteomic and genomic analyses of antimony resistant leishmania infantum mutant. PLoS One.

[18] Tschoeke DA, Nunes GL, Jardim R, Lima J, Dumaresq AS, Gomes MR, de Mattos PL, Loureiro DR, Stoco PH, de Matos Guedes HL, de Miranda AB, Ruiz J, Pitaluga A, Silva FP, Probst CM, Dickens NJ, Mottram JC, Grisard EC, Dávila AM (2014). The comparative genomics and phylogenomics of leishmania amazonensis parasite. Evol Bioinforma Online.

[19] Hardison RC (2003). Comparative genomics. PLoS Biol.

[20] Koonin EV (2005). Orthologs, paralogs, and evolutionary genomics. Annu Rev Genet.

[21] Sequence - Evolution - Function - NCBI Bookshelf [http://www.ncbi.nlm.nih.gov/books/NBK20260/].

[22] Dessimoz C, Gabaldón T, Roos DS, Sonnhammer ELL, Herrero J, Quest for Orthologs Consortium (2012). Toward community standards in the quest for orthologs. Bioinforma Oxf Engl.

[23] Delsuc F, Brinkmann H, Philippe H (2005). Phylogenomics and the reconstruction of the tree of life. Nat Rev Genet.

[24] Li L, Stoeckert CJ, Roos DS (2003). OrthoMCL: Identification of ortholog groups for eukaryotic genomes. Genome Res.

[25] Dessimoz C (2011). Editorial: Orthology and applications. Brief Bioinform.

[26] Kristensen DM, Wolf YI, Mushegian AR, Koonin EV (2011). Computational methods for Gene Orthology inference. Brief Bioinform.

[27] Cuadrat RRC, Cruz SM da S, Tschoeke DA, Silva E, Tosta F, Jucá H, et al. An Orthology-Based Analysis of Pathogenic Protozoa Impacting Global Health: An Improved Comparative Genomics Approach with Prokaryotes and Model Eukaryote Orthologs. OMICS J Integr Biol. 2014;140624130015005. 10.1089/omi.2013.0172 PMC4108940 24960463

[28] Dewey CN (2011). Positional orthology: putting genomic evolutionary relationships into context. Brief Bioinform.

[29] Dalquen DA, Altenhoff AM, Gonnet GH, Dessimoz C (2013). The Impact of Gene Duplication, Insertion, Deletion, Lateral Gene Transfer and Sequencing Error on Orthology Inference: A Simulation Study. PLoS One.

[30] Da Cruz SMS, Batista V, Silva E, Tosta F, Vilela C, Cuadrat R, Tschoeke D, Dávila AMR, Campos MLM, Mattoso M (2010). Detecting distant homologies on protozoans metabolic pathways using scientific workflows. Int J Data Min Bioinforma.

[31] Remm M, Storm CEV, Sonnhammer ELL (2001). Automatic clustering of orthologs and in-paralogs from pairwise species comparisons. J Mol Biol.

[32] DeLuca TF, Cui J, Jung J-Y, Gabriel KCS, Wall DP (2012). Roundup 2.0: Enabling comparative genomics for over 1800 genomes. Bioinformatics.

[33] Wall DP, Fraser HB, Hirsh AE (2003). Detecting putative orthologs. Bioinformatics.

[34] Rasmussen MD, Kellis M (2011). A Bayesian Approach for Fast and Accurate Gene Tree Reconstruction. Mol Biol Evol.

[35] Chen F, Mackey AJ, Stoeckert CJ, Roos DS (2006). OrthoMCL-DB: querying a comprehensive multi-species collection of ortholog groups. Nucleic Acids Res.

[36] O’Brien KP, Remm M, Sonnhammer ELL (2005). Inparanoid: a comprehensive database of eukaryotic orthologs. Nucleic Acids Res.

[37] Tatusov RL, Fedorova ND, Jackson JD, Jacobs AR, Kiryutin B, Koonin EV, Krylov DM, Mazumder R, Mekhedov SL, Nikolskaya AN, Rao BS, Smirnov S, Sverdlov AV, Vasudevan S, Wolf YI, Yin JJ, Natale DA (2003). The COG database: an updated version includes eukaryotes. BMC Bioinformatics.

[38] Powell S, Forslund K, Szklarczyk D, Trachana K, Roth A, Huerta-Cepas J, et al. eggNOG v4.0: nested orthology inference across 3686 organisms. Nucleic Acids Res 2013;42(D1):gkt1253. 10.1093/nar/gkt1253 PMC3964997 24297252

[39] Powell S, Szklarczyk D, Trachana K, Roth A, Kuhn M, Muller J, Arnold R, Rattei T, Letunic I, Doerks T, Jensen LJ, von Mering C, Bork P (2011). eggNOG v3.0: orthologous groups covering 1133 organisms at 41 different taxonomic ranges. Nucleic Acids Res.

[40] Da Cruz SMS, Batista V, Dávila AMR, Silva E, Tosta F, Vilela C, Campos MLM, Cuadrat R, Tschoeke D, Mattoso M (2008). OrthoSearch: a scientific workflow approach to detect distant homologies on protozoans.

[41] Taylor IJ, Deelman E, Gannon DB, Shields M (2007). Workflows for E-Science.

[42] Timmers LFSM, Pauli I, Barcellos GB, Rocha KB, Caceres RA, de Azevedo WF, Soares MBP (2009). Genomic databases and the search of protein targets for protozoan parasites. Curr Drug Targets.

[43] S SK, R.K. G, Ghosh M (2014). Comparative in-silico genome analysis of Leishmania (Leishmania) donovani: A step towards its species specificity. Meta Gene.

[44] KEGG Orthology [http://www.genome.jp/kegg/ko.html].

[45] KEGG. Kyoto Encyclopedia of Genes and Genomes [http://nar.oxfordjournals.org/content/28/1/27.abstract?ijkey=8863b3d5385d8722cdd87f19eccd8a62b567a0b4&keytype2=tf_ipsecsha].

[46] Dávila AMR, Mendes PN, Wagner G, Tschoeke DA, Cuadrat RRC, Liberman F, Matos L, Satake T, Ocaña KACS, Triana O, Cruz SMS, Jucá HCL, Cury JC, Silva FN, Geronimo GA, Ruiz M, Ruback E, Silva FP, Probst CM, Grisard EC, Krieger MA, Goldenberg S, Cavalcanti MCR, Moraes MO, Campos MLM, Mattoso M (2008). ProtozoaDB: dynamic visualization and exploration of protozoan genomes. Nucleic Acids Res.

[47] Camacho C, Coulouris G, Avagyan V, Ma N, Papadopoulos J, Bealer K, Madden TL (2009). BLAST+: architecture and applications. BMC Bioinformatics.

[48] RefSeq: NCBI Reference Sequence Database [http://www.ncbi.nlm.nih.gov/refseq/].

[49] Ashburner M, Ball CA, Blake JA, Botstein D, Butler H, Cherry JM, Davis AP, Dolinski K, Dwight SS, Eppig JT, Harris MA, Hill DP, Issel-Tarver L, Kasarskis A, Lewis S, Matese JC, Richardson JE, Ringwald M, Rubin GM, Sherlock G (2000). Gene Ontology: tool for the unification of biology. Nat Genet.

[50] Chen F, Mackey AJ, Vermunt JK, Roos DS. Assessing Performance of Orthology Detection Strategies Applied to Eukaryotic Genomes. PLoS One. 2007;2. 10.1371/journal.pone.0000383 PMC1849888 17440619

[51] McCormack JE, Faircloth BC, Crawford NG, Gowaty PA, Brumfield RT, Glenn TC (2012). Ultraconserved elements are novel phylogenomic markers that resolve placental mammal phylogeny when combined with species-tree analysis. Genome Res.

[52] Konstantinidis KT, Tiedje JM (2005). Genomic insights that advance the species definition for prokaryotes. Proc Natl Acad Sci.

[53] Colotti G, Baiocco P, Fiorillo A, Boffi A, Poser E, Chiaro FD, Ilari A (2013). Structural insights into the enzymes of the trypanothione pathway: targets for antileishmaniasis drugs. Future Med Chem.

[54] Krauth-Siegel RL, Meiering SK, Schmidt H. The Parasite-Specific Trypanothione Metabolism of Trypanosoma and Leishmania. Biol Chem. 2003;384. 10.1515/BC.2003.062 12751784

[55] Shinde S, Mol M, Jamdar V, Singh S (2014). Molecular modeling and molecular dynamics simulations of GPI 14 in Leishmania major: insight into the catalytic site for active site directed drug design. J Theor Biol.

[56] Garami A, Ilg T (2001). The role of phosphomannose isomerase in Leishmania mexicana glycoconjugate synthesis and virulence. J Biol Chem.

[57] Ito M, Guffanti AA, Krulwich TA (2001). Mrp-dependent Na+/H+ antiporters of Bacillus exhibit characteristics that are unanticipated for completely secondary active transporters. FEBS Lett.

[58] Hiramatsu T, Kodama K, Kuroda T, Mizushima T, Tsuchiya T (1998). A putative multisubunit Na+/H+ antiporter from Staphylococcus aureus. J Bacteriol.

[59] Huang C, Turco SJ (1993). Defective galactofuranose addition in lipophosphoglycan biosynthesis in a mutant of Leishmania donovani. J Biol Chem.

[60] Zhang K, Barron T, Turco SJ, Beverley SM (2004). The LPG1 gene family of Leishmania major. Mol Biochem Parasitol.

[61] Spath GF, Epstein L, Leader B, Singer SM, Avila HA, Turco SJ, Beverley SM (2000). Lipophosphoglycan is a virulence factor distinct from related glycoconjugates in the protozoan parasite Leishmania major. Proc Natl Acad Sci.

